# Difficult diagnosis in the clinical evaluation of a patient with squamous cell carcinoma of the sigmoid colon: a case report

**DOI:** 10.1186/s13256-023-04051-4

**Published:** 2023-07-29

**Authors:** Alonso Garro-Mendiola, David Guevara-Lazo, Willian Pinto Samanez, Juan Carlos Lizarzaburu-Robles

**Affiliations:** 1 Universidad Científica del Sur, Lima, Perú; 2 Universidad Peruana Cayetano Heredia, Lima, Perú; 3 Internal Medicine Department, ESSALUD, Moquegua, Perú; 4 Escuela de Doctorado, Universidad Autónoma de Madrid, Madrid, Spain

**Keywords:** Colorectal cancer, Early intervention, Primary care, Squamous cell carcinoma

## Abstract

**Background:**

Squamous cell carcinoma (SCC) of the sigmoid colon is an exceedingly rare subtype of colorectal cancer (CRC) associated with chronic inflammatory conditions. Due to its variable clinical presentation ranging from subclinical to fully symptomatic and limited available information, it poses a diagnostic challenge. We aim to provide a review of the current literature and raise awareness about the importance of a thorough clinical analysis for an early diagnosis.

**Case presentation:**

We describe the case of a 59-year-old Peruvian woman with a medical history of diverticular disease and irritable bowel syndrome. The patient presented with nonspecific symptoms such as abdominal discomfort, constipation, and bloating. Diagnostic tests and biopsy revealed a rare case of squamous cell carcinoma of the sigmoid colon. The patient underwent surgical resection and adjuvant chemotherapy.

**Conclusion:**

Despite the rarity of this type of cancer in the colon, the patient's clinical course highlights the importance of considering it as a potential diagnosis in patients with nonspecific symptoms and a history of gastrointestinal disorders. Surgical treatment followed by radiotherapy is the preferred management. Factors such as lack of postoperative complications and the stage of the neoplasia can augur a positive. prognosis. A prompt diagnosis is crucial, as detecting a neoplasia in its early stages can make surgery more effective.

## Background

Primary squamous cell carcinoma (SCC) is a rare histological subtype of Colorectal Cancer (CRC) with an approximate incidence of 0.10 to 0.25 per 1000 colorectal cancers. It most commonly occurs in the right colon and least frequently in the sigmoid colon. The clinical presentation of CRC is similar regardless of histological subtype and can range from classical gastrointestinal bleeding, intestinal obstruction and weight loss to an asymptomatic patient [1]. We present this unique case to raise awareness about the importance of a detailed clinical evaluation for an early diagnosis.

## Clinical case presentation

A 59-year-old Peruvian female presented to the outpatient clinic with chronic abdominal pain and bloating. Medical history was relevant for obesity (Body Mass Index (BMI) 31 kg/m^2^), diverticular disease and irritable bowel syndrome (IBS). Her familiar history was significant for Type 2 Diabetes Mellitus and hypertension. Previous hospitalizations were related to pelvic inflammatory disease and complicated diverticulitis. Her regular medications were fiber supplementation and simethicone.

The physical examination revealed mild abdominal tenderness in the left lower quadrant without associated fever or change in bowel habits. Abdominal imaging and routine laboratory exams were performed. The abdominal ultrasound revealed an enlarged uterus reported as uterine myomatosis. Due to the non-specific clinical features, unrevealing imaging and past medical history, an IBS flare was suspected and supportive medication was prescribed.

**Fig. 1:**
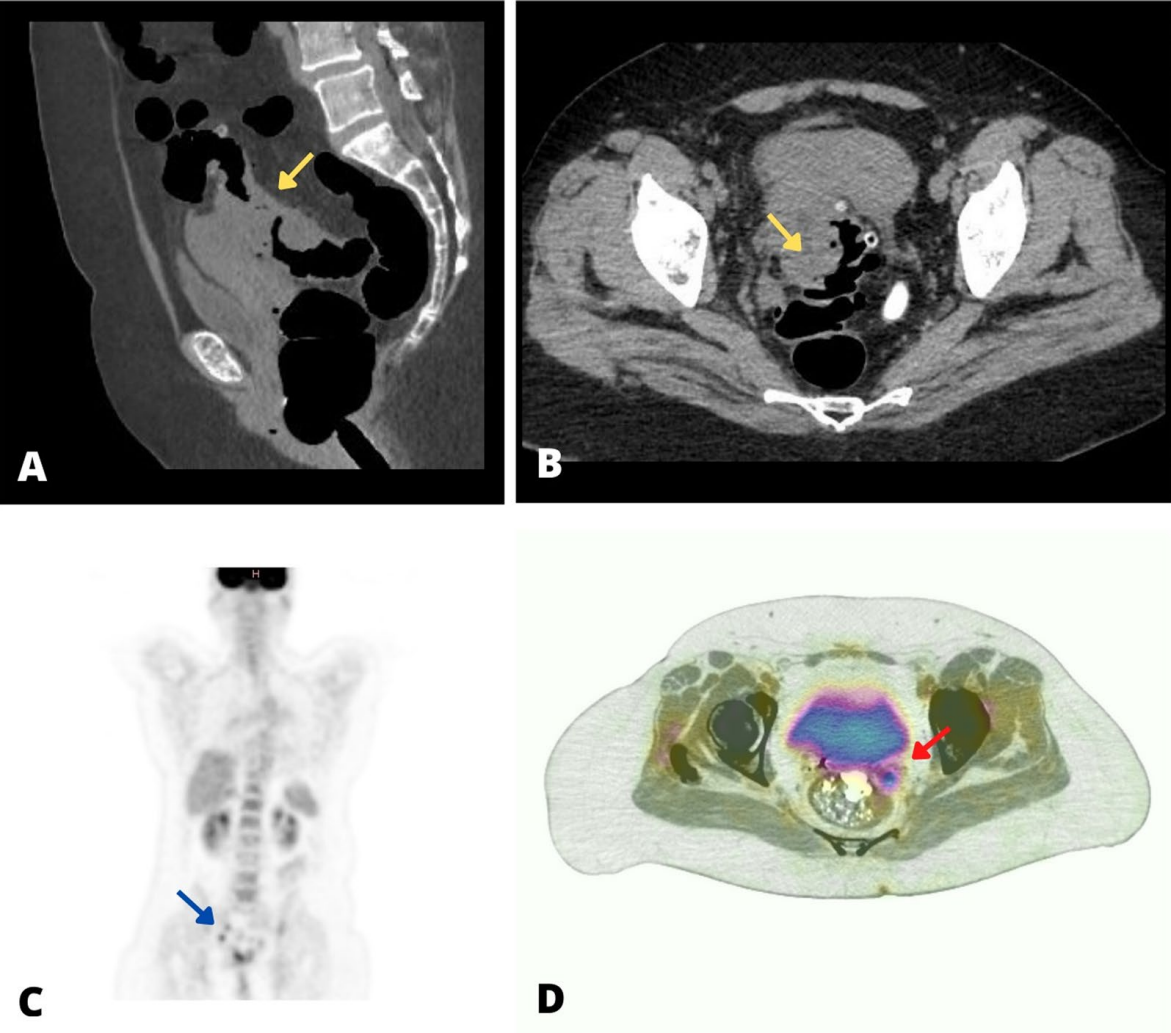
Advanced Imaging. Panels **A** and **B** are computed tomography showing the primary lesion involving the uterus and lumen of the colon (yellow arrow). Panel **C** is the first Positron Emission Tomography and Computed Tomography (PET-CT) scan showing two hypermetabolic nodules (< 1 cm) near the right lymphatic iliac chain (blue arrow). Panel **D** is the second PET-CT ordered after the second chemotherapeutic regimen, revealing a hypermetabolic nodular (2 × 2 cm) lesion behind the bladder near the sigmoid colon (red arrow)

A week later, the patient presented to the gynecology outpatient clinic due to persistent symptoms. A transvaginal ultrasound found a mass near the left ovary. The differential diagnoses were ovarian and CRC. A virtual colonoscopy was ordered because extraluminal compression caused an incomplete colonoscopic evaluation. Thoracoabdominal Magnetic Resonance Imaging (MRI) and tumor markers (Cancer Antigen 125 (CA-125) and Carcinoembryonic Antigen (CEA)) were performed. The CEA was elevated tenfold its normal value and the MRI revealed a large 9 cm tumoral lesion. stenosing the sigmoid lumen with attachments to the left ovary and uterus (Fig. 1A, B), no thoracic metastatic lesions were found. Virtual colonoscopic evaluation was not contributory.

**Fig. 2:**
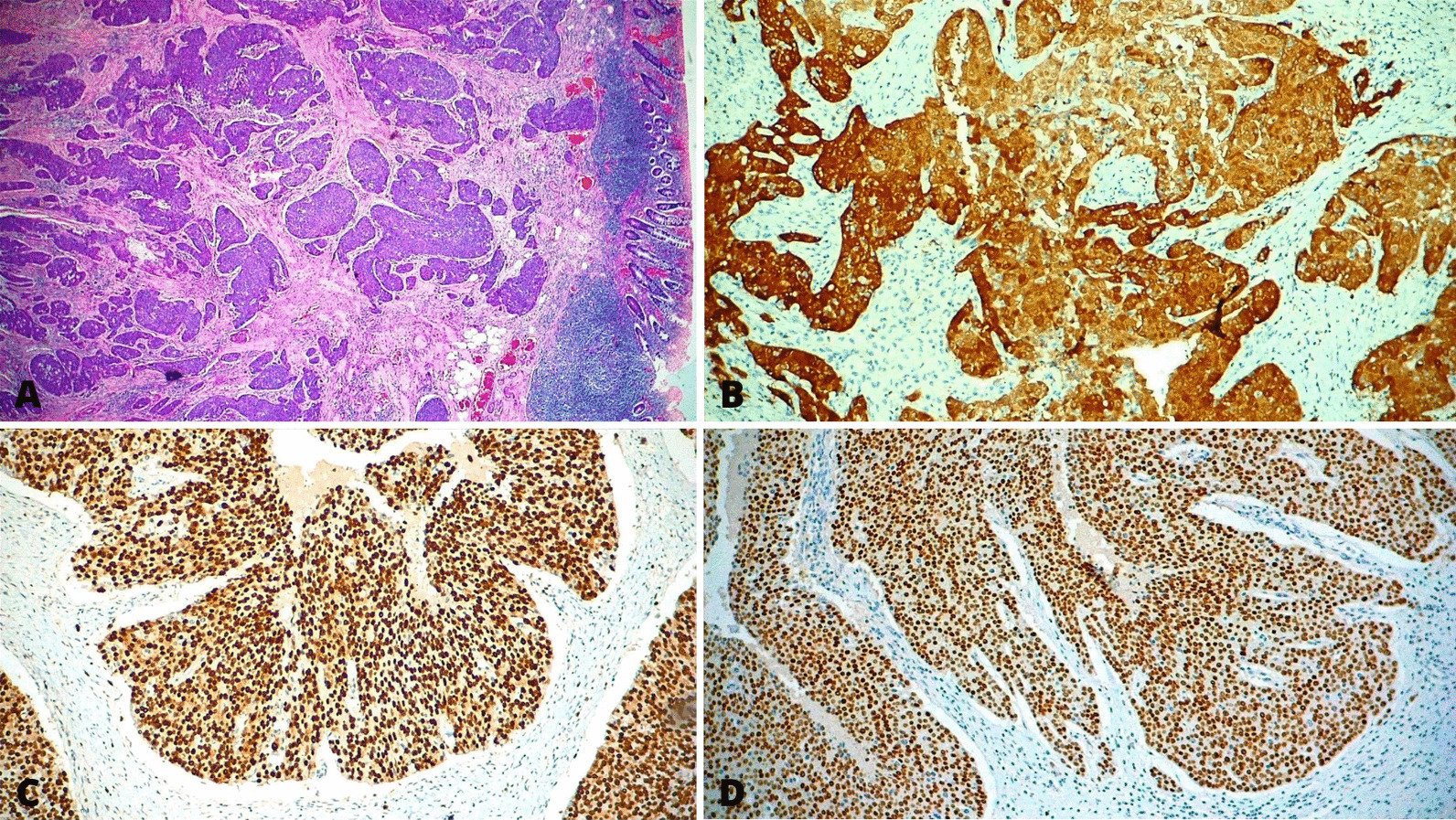
Histology slides. The panels **A** and **B** are Hematoxylin and Eosin (HE) stains at magnification of 2.5X and 40X respectively. They are consistent with poorly differentiated squamous cell carcinoma, panel **A** shows tumor invasion of the muscular and submucosal layer. On panel **B** we see pleomorphic and abnormal mitotic cells arranged in an irregular pattern. Panels **C** and **D** are immunohistochemical analysis positive for P16 (40x) and P40 (10x) stains respectively

Surgical treatment was decided due to a high suspicion of neoplasia. A laparoscopic left hemicolectomy, hysterectomy and bilateral salpingo-oophorectomy were performed along with intraoperative ultrasound showing the absence of metastatic lesions in abdominal organs. Forty-eight lymphatic nodes were analyzed without neoplastic infiltration. No complications were reported in the procedure. A diagnosis of primary SCC of the colon was confirmed on histopathological and immunohistochemical analysis (Fig. 2).

Immunohistochemistry showed overexpression of p16, p40 and ki67, subsequent Polymerase Chain Reaction (PCR) analysis for Human Papillomavirus (HPV) on tissue samples were performed, yielding a negative result. Genomic profiling demonstrated stable microsatellite status, mutations in Phosphatase and Tensin Homolog (PTEN), Anaplastic Lymphoma Kinase (ALK), Fas cell surface death receptor (FAS) and Tumor Protein p53 (TP53). The stage of the neoplasia was T4b, N0, M0 (Stage IIc).

### Follow-up

Three months after surgery, a PET-CT scan revealed two hypermetabolic nodules (Fig. 1C). Post-surgical inflammatory nodules versus neoplastic recurrence were considered, conservative management with adjuvant chemotherapy was indicated for 4 months with Carboplatin (300 mg) and Paclitaxel (300 mg).

After the first chemotherapeutic regimen, a CT-scan showed that the previous nodules had increased in size (3 × 3 cm), infiltrating the cecum and the right ureter. A right hemicolectomy and ureteral sectioning plus anastomosis was performed without complications. Histopathological analysis confirmed recurrence.

Adjuvant chemotherapy with Cisplatin (100 mg) and 5-fluorouracil (1200 mg) infusion for 6 months was administered. A PET-CT scan after the second chemotherapeutic regimen revealed a hypermetabolic nodular lesion (Fig. 1D). Neoplastic recurrence was likely, and a third surgery was performed to remove the mass. There were no postoperative complications. Histopathological analysis confirmed recurrence. Daily radiotherapy for 30 days was indicated. Currently, the patient is free of active disease.

## Discussion

### Physiopathology

Several hypotheses have been proposed for the development of CRC. The most accepted ones are the adenoma-carcinoma sequence and the serrated-neoplasia sequence. Where an adenoma accumulates genetic and epigenetic mutations in its progression to a neoplasm. The adenoma-carcinoma pathway starts with a mutation in the genes APC, RAS or TP53. On the other hand, the serrated-neoplasia pathway is associated with mutations in the oncogenes Neuroblastoma RAS Viral Oncogene Homolog (NRAS) and B-Raf proto-oncogene (BRAF). In this case the genomic profiling demonstrated KRAS, BRAF wild-type and mutations in PTEN, ALK, FAS, TP53 genes which opens-up a new layer of treatment options [2].

Since SCC is a subtype of CRC, additional etiologies have been suggested, multipotent stem cells of the colonic mucosa could undergo metaplasia and differentiate into squamous epithelium due to chronic inflammation, such as inflammatory bowel disease, obesity, chronic infections, complicated diverticular disease, among others. The past history of inflammatory conditions of our patient may have increased her risk of developing a gastrointestinal neoplasia [1].

### Diagnosis

Four criteria must be met to diagnose colorectal SCC. Firstly, there should be no evidence of SCC in any other organ that might spread or metastasize to the bowel. Secondly, a squamous-lined fistula between the bowel and a site of origin of the SCC should be excluded. Thirdly, in the case of SCC of the rectum, the extension of a tumor arising from the anal squamous epithelium should be ruled out. Fourthly, histological analysis must confirm the SCC, a positive p40 stain (Fig. 2D) is highly sensitive and specific for squamous cell carcinoma [3]. Our case fulfills all four criteria.

The patient's past medical history, non-specific symptoms and ultrasound report made the diagnosis difficult, highlighting the importance of clinical-radiological correlation [4]. The diagnosis of an enlarged uterus due to myomatosis by abdominal ultrasound in a postmenopausal woman who is not receiving hormonal replacement therapy was unlikely. This should broaden the differential diagnosis and lower the threshold for advanced imaging (CT, MRI). In addition, it enables surgical planning and evaluation of tumor extension. High-risk patients with symptoms refractory to treatment should raise suspicion for CRC.

### Treatment

Colorectal SCC is treated like colorectal adenocarcinoma due to its low incidence and lack of information. Currently, case reports and a systematic review consider surgery as the mainstay of treatment, with chemotherapy and radiotherapy reserved for non-surgical candidates [1]. However, poor outcomes have been reported in patients treated solely with chemotherapy. Immunotherapy is being studied as an alternative for neoplasias refractory to chemotherapy, with the choice of biologic-agent tailored to the genetic characteristics of the neoplasia, which are unique in every patient. Cetuximab and Panitumumab were the available agents for our patient.

### Prognosis

Due to the limited information about the disease, prognostic evaluation becomes difficult. According to a recent systematic review and meta-analysis, factors strongly associated with a poor prognosis are Tumor (4b) Nodule (0) Metastasis (0) (TNM) stage ≥ 3, postoperative complications and p16 overexpression (1, 5). The patient had no postoperative complications, a TNM of 2 and overexpressed p16 in tumoral cells. P16 protein overexpression (Fig. 2D) is significantly associated with risk of CRC and is a useful biomarker to predict disease progression (TNM-stage, Dukes stage, lymph node metastasis). However, further studies should be made to further characterize the influence of p16 expression in CRC (5).

## Conclusion

SCC of the sigmoid is best treated with surgery and radiotherapy. Unexpected findings in the clinical evaluation should lower the threshold for advanced imaging (CT) in patients with nonspecific gastrointestinal symptoms. Early detection is key for a favorable prognosis, since the stage of the neoplasia determines how effective surgery can be.

## Data Availability

Not applicable.
